# Friedelin: Structure, Biosynthesis, Extraction, and Its Potential Health Impact

**DOI:** 10.3390/molecules28237760

**Published:** 2023-11-24

**Authors:** Santosh Kumar Singh, Shweta Shrivastava, Awdhesh Kumar Mishra, Darshan Kumar, Vijay Kant Pandey, Pratima Srivastava, Biswaranjan Pradhan, Bikash Chandra Behera, Ashutosh Bahuguna, Kwang-Hyun Baek

**Affiliations:** 1 Department of Biotechnology, ARKA Jain University, Jamshedpur 832108, Jharkhand, India; dr.santosh@arkajainuniversity.ac.in (S.K.S.); dr.pratima@arkajainuniversity.ac.in (P.S.); 2 School of Pharmacy, ARKA Jain University, Jamshedpur 832108, Jharkhand, India; dr.shweta@arkajainuniversity.ac.in; 3 Department of Biotechnology, Yeungnam University, Gyeongsan 38541, Republic of Korea; 4 Department of Agriculture, Netaji Subhas University, Jamshedpur 831012, Jharkhand, India; pandeyvijay00@gmail.com; 5 S.K. Dash Centre of Excellence of Biosciences and Engineering and Technology, Indian Institute of Technology, Bhubaneswar 752050, Odisha, India; bp21@iitbbs.ac.in; 6 School of Biological Sciences, National Institute of Science Education and Research, Bhubaneswar 752050, Odisha, India; bikash@niser.ac.in; 7 Department of Food Science and Technology, Yeungnam University, Gyeongsan 38541, Republic of Korea; ashubahuguna@gmail.com

**Keywords:** friedelin, phytochemicals, extraction methods, anticancer, neuroprotective, antimicrobial

## Abstract

Pharmaceutical companies are investigating more source matrices for natural bioactive chemicals. Friedelin (friedelan-3-one) is a pentacyclic triterpene isolated from various plant species from different families as well as mosses and lichen. The fundamental compounds of these friedelane triterpenoids are abundantly found in cork tissues and leaf materials of diverse plant genera such as Celastraceae, Asteraceae, Fabaceae, and Myrtaceae. They possess many pharmacological effects, including anti-inflammatory, antioxidant, anticancer, and antimicrobial activities. Friedelin also has an anti-insect effect and the ability to alter the soil microbial ecology, making it vital to agriculture. Ultrasound, microwave, supercritical fluid, ionic liquid, and acid hydrolysis extract friedelin with reduced environmental impact. Recently, the high demand for friedelin has led to the development of CRISPR/Cas9 technology and gene overexpression plasmids to produce friedelin using genetically engineered yeast. Friedelin with low cytotoxicity to normal cells can be the best phytochemical for the drug of choice. The review summarizes the structural interpretation, biosynthesis, physicochemical properties, quantification, and various forms of pharmacological significance.

## 1. Introduction

Plants in the form of whole plants, vegetables, fruits, whole grains, and nuts provide various phytochemicals like phenolic compounds, terpenoids, alkaloids, pigments, and other natural antioxidants [1,2]. These phytochemicals are nonnutritive substances that possess large health-protective benefits [3]. Over 80% of the world’s population relies on the traditional medical system to treat their health issues [4].

Friedelin (friedelan-3-one) is a pentacyclic triterpene first isolated from bark in 1807 using alcohol and called “cork alcohol” [5]. Later it was isolated from various plant species from different families [6,7,8] and also reported to isolate from lower plants like mosses [9], lichen [10,11], algae [12], and fungi [13]. In recent years, a substantial number of research studies have demonstrated extraordinary pharmacological actions of friedelin such as anti-inflammatory [14], anticancer [15,16,17,18], antioxidant and hepatoprotective [19,20,21], neuroprotective [22,23], antimicrobial [24,25,26], and antidiabetic effects [21,27,28]. Further, friedelin has outstanding natural anti-insect action, especially against *S. litura* and *H. armigera* [29], and could modulate the dynamics of the soil microbial community [30] and thus play an important role in agriculture. Friedelin has also been suggested for use as an herbicide due to the fact that it inhibits photosynthesis. [31,32] found that its phytotoxic activity inhibited both the root and shoot germination of wheat, rice, and pea germinating seeds. Consequently, friedelin and its derivatives offer prospective resources for the creation of novel medications or nutritional supplements.

Initially, the compound was extracted by soaking plant materials in various organic solvents. However, with the advancement of technology, modern extraction and analytical methods for the extraction of friedelin from different plant materials were used. With its great pharmaceutical usage, the amount of friedelin that can be obtained from plant sources is insufficient, whilst the amount that can be obtained through chemical processes is sometimes difficult to achieve, requires intense reaction conditions, and results in the production of unsafe compounds. In 2022, Wang et al. [33] used CRISPR/Cas9 technology and gene overexpression plasmids to produce friedelin using genetically engineered yeast.

According to the information obtained from various kinds of published research, friedelin could be a potential source in preventing various chronic diseases after the extraction and incorporation are completed at the target location. Nevertheless, the production of friedelin involves a number of significant procedures, the most important of which are the identification of the source, the determination of an appropriate quantity, and the execution of an appropriate extraction method. Therefore, this study is a complete presentation addressing the sources, qualities, and applications of friedelin, with a main emphasis on its numerous extraction procedures along with their applicability, limitations, and prospective remedies.

## 2. Sources of Friedelin

Due to the adverse effects of modern medications and therapies, plant products as therapeutic options are used worldwide. Friedelin has been studied for its potential biological activities and medicinal properties. One of the most significant natural sources of friedelin is found in cork. In the form of powder or granules, both natural and treated cork/bark are said to contain a large amount of friedelin. Leaves that are used with food directly are another source of friedelin. Friedelin was isolated from a wide variety of plant genera that belong to a wide range of plant families, and it was also found to be isolated from lower plants such as mosses [9], lichen [10], and algae [12]. *Calophyllum pinetorum*, *Garcinia prainiana, Garcinia imberti*, *Garcinia rubroechinata,* and *Mammea siamensis* belonging to the Clusiaceae family are major sources of friedelin. *Drypetes tessmanniana*, *Putranjiva roxburghii, Uapaca ambanjensis,* and *Euphorbia tirucalli* L. belonging to the family Euphorbiaceae; *Salix tetrasperma* and *Populus davidiana* belonging to the family Salicaceae; *Terminalia avicennioides* and *Combretum duarteanum* of the family Combretaceae; *Luehea ochrophylla* and *Ancistrocarpus densispinosus* of the family *Tiliaceae; Prunus turfosa* and *Prunus lusitanica* of the family Rosaceae and *Maytenus ilicifolia* and *Maytenus aquifolium* of family Celastraceae have been reported for the extraction of friedelin (Table 1).

## 3. Chemistry and Biosynthesis Pathway of Friedelin

Triterpenes are an essential class of chemicals that are present in both prokaryotes and eukaryotes and are involved in a wide variety of biological processes [77]. Triterpenoids and their derivatives have been shown to have a variety of functions, including those of hormones [78], lipid membrane components [79], and defense chemicals [80,81,82]. In addition, numerous triterpenoids, or their downstream products, have medical applications [83,84,85]. The biosynthesis of triterpenoids begins with the oxidation of 2-3-oxidosqualene, which is followed by protonation, cyclization, and several rearrangements [77]. This substance is capable of being transformed into a large variety of structurally distinct backbones, with over 100 of them having been found in plants alone. Members of the oxidosqualene cyclases (OSCs) and squalene-hopene cyclases (SHCs) families of enzymes commonly carry out these changes [77].

**Figure 1:**
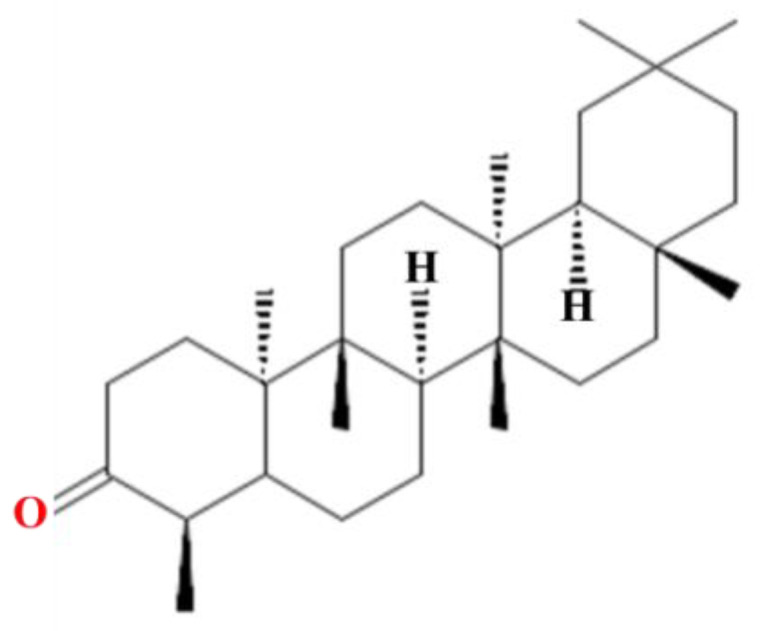
Structure of friedelin with UPAC name (4R,4aS,6aS,6aS,6bR,8aR,12aR,14aS,14bS)-4,4a,6a,6b,8a,11,11,14a-octamethyl-2,4,5,6,6a,7,8,9,10,12,12a,13,14,14b-tetradecahydro-1H-picen-3-one.

Friedelin is a pentacyclic triterpenoid that is derived from perhydropicene [45]. There is a substituent of an oxo group at position 3 of the perhydropicene molecule, as well as methyl groups at positions 4, 4a, 6b, 8a, 11, 11, 12b, and 14a [45]. It has a pentacyclic structure consisting of five rings with the molecular formula C_30_H_50_O (Figure 1). It is an extremely basic compound with a pKa value of −7.4 and showed high solubility in chloroform, sparing solubility in ethanol, and insolubility in water [40]. The molecular weight of friedelin is 426.7 g/mol with a topological surface area of 17.1 Å^2^. It contains one hydrogen bond acceptor, nine defined atom stereocenters, and one covalently bonded unit (details of chemical and physical properties of friedelin are shown in Table S1).

**Figure 2:**
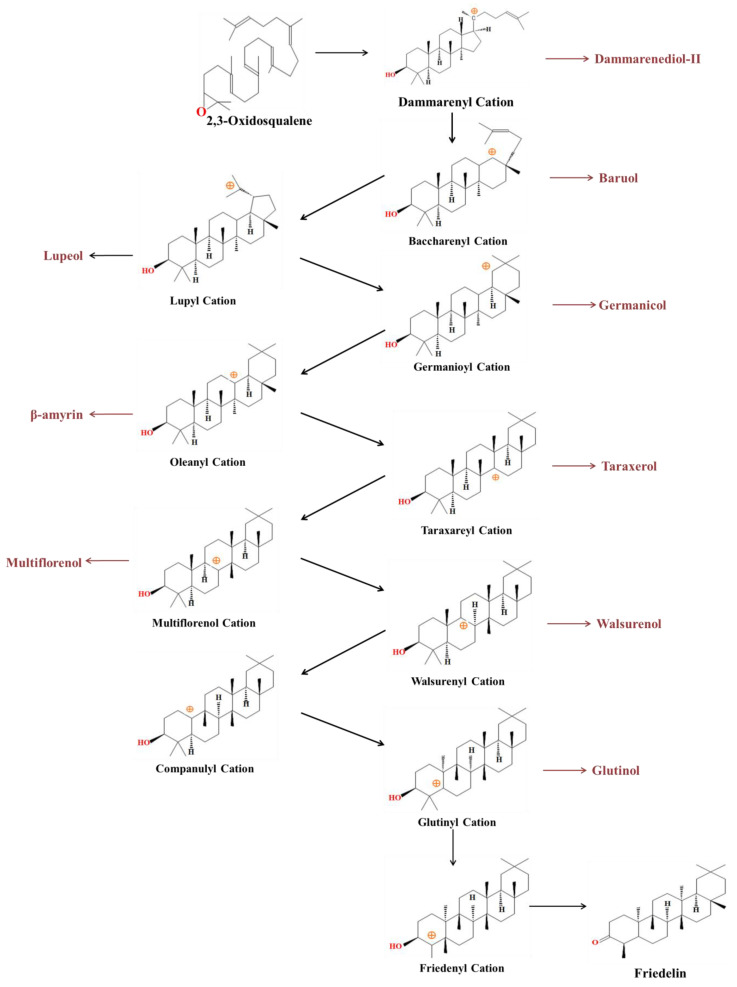
Pentacyclic friedelin biosynthesis from 2, 3-oxidosqualene through oxidosqualene protonation, cyclization, several rearrangements, and deprotonating.

The biosynthesis of friedelin is a continuous process involving the pentacyclization of squalene oxide to the lupanyl cation and ten suprafacial 1,2-shifts of methyls and hydrogens as depicted in Figure 2. The first phase starts with the condensation of acetyl coenzyme A (CoA) units catalyzed by acetyl-CoA C-acetyltransferase (AACT) to produce acetoacetyl CoA. Subsequently, 3-hydroxy-3-methylglutaryl CoA (HMGCoA) synthase converts acetoacetyl CoA to hydroxy-3-methylglutaryl-CoA, which is regarded as a precursor to cholesterol. NADPH-dependent HMGCoA reductase converts HMGCoA into mevalonate (MVA). A series of enzymes then convert MVA into isopentenyl pyrophosphate (IPP), the five-carbon isoprenoid unit. IPP isomerase can convert IPP into dimethylallyl pyrophosphate (DMAPP) in a reversible manner. IPP and DMAPP are regarded as substrates shared by both pathways. DMAPP molecules are converted into farnesyl diphosphate by the enzyme farnesyl pyrophosphate synthase followed by squalene by the enzyme squalene synthase and finally to 2,3-Oxidosqualene by the enzyme squalene epoxidase [79].

In the second phase, oxidosqualene is protonated by oxidosqualene cyclases followed by cyclization, rearrangements, and deprotonation to synthesis friedelin [51,86]. Further, oxidosqualene cyclization begins with carbocation, which undergoes multiple rearrangements to generate various compounds. In the pathway, 2, 3-oxidosqualene converted into dammarenyl cation followed by baccharenyl cation, lupyl cation, Germanioyl cation, oleamyl cation, taraxaeyl cation, multiflorenol cation, Walsurenyl cation, companulyl cation, glutinyl cation, friedenyl cation, and finally friedelin (Figure 2).

## 4. Extraction Methods and Quantification

### 4.1. Extraction Methods and Analysis

The process of phytochemical extraction and purification from plant material is typically influenced by a number of parameters, the most important of which are time, temperature, the concentration of the solvent, and the polarity of the solvent [87]. Because it is unlikely that a single solvent could reliably extract all of the phytochemicals present in the plant material, distinct phytochemicals are extracted in solvents of varying polarity according to the chemical nature of the phytochemical.

**Figure 3:**
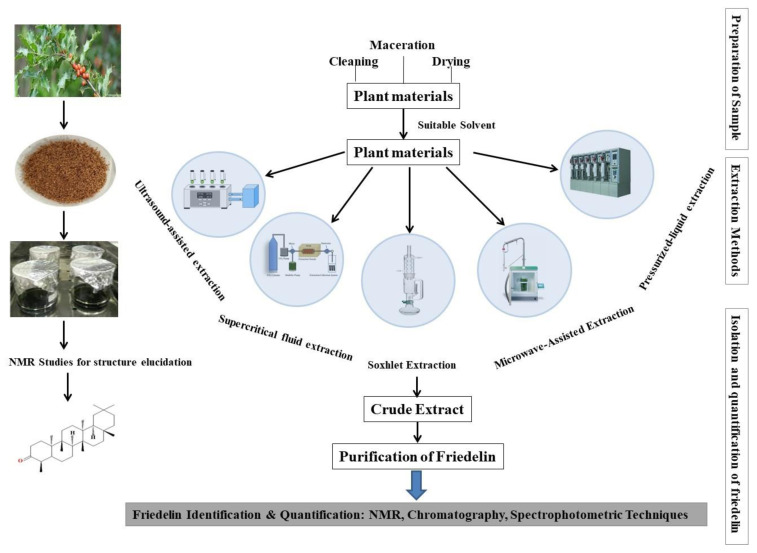
Extraction methods of friedelin from different plant materials.

Friedelin is a pentacyclic triterpenoid non-polar compound. Various extraction methods reported to be used for friedelin extraction are represented in Figure 3. Depending upon the plant parts used, organic solvents such as methanol, ethanol, hexane, dichloromethane, petroleum ether, and chloroform are commonly used to extract friedelin (aforementioned Table 1). Soxhlet has been the most common extraction method of friedelin for a very long time (Table 2). This assertion is supported by the fact that Soxhlet has been a standard technique for more than a century and is currently the standard against which other leaching methods are measured [88].

Supercritical fluid extraction (SFE) is an environmentally friendly and selective method to obtain plant extracts [91,92]. de Vasconcelos et al. [90] used a modified supercritical fluid extraction-CO_2_ method in which plant material was extracted with CO_2_ modified with methanol (10% *v*/*v*), ethanol (10% *v*/*v*), or pentane (10% or 20% *v*/*v*). They found that SFE CO_2_ modified with 10% methanol and SFE CO_2_ modified with 10% ethanol had the highest concentration of friedelin with 6.10 ± 0.75 mg/g plant material and 6.00 ± 0.97 mg/g plant material, respectively, as compared to SFE CO_2_ modified with 10% pentane, which had a friedelin yield of 3.00 ± 0.90 mg/g plant material, whereas SFE CO_2_ modified with 20% pentane had a friedelin yield of 3.70 ± 0.98 mg/g plant material [90]. De Melo et al. [86] used pure and ethanol-modified CO_2_ for the extraction of friedelin by the supercritical fluid extraction method. Different particle size (coarse particles to >80 mesh size) and ethanol (CO_2_ modifier) content (0–5 wt.%) in a 0.5 L capacity unit, at 300 bar, 50 ºC, and a CO_2_ flow rate of 11 g/min was used for the total (η_total_) and friedelin (η_friedelin_) extraction. In this extraction method, they found that intermediate granulometries (40–60 mesh to 60–80 mesh) and a CO_2_: EtOH ratio 95.0: 5.0 wt% with 300 bar pressure enhance the friedelin selectivity by up to 2.6 times [86].

Vieira et al. [34] used a supercritical fluid extraction method for the extraction of friedelin from *Quercus cerris* bark. They analyzed two CO_2_:EtOH ratios, 95.0:5.0 wt% and 97.5:2.5 wt%, with Q_CO2_ 5 g/min & 8 g/min and temperature ranges 40–60 °C at a constant pressure of 300 bar. They found that the CO_2_:EtOH ratio of 97.5:2.5 wt%, Q_CO2_ 8 g/min, and temperature 60 °C had the highest extraction yield of 0.48 wt% with a friedelin concentration of 28 wt% [34]. Kornpointner et al. [65] used this extraction method with a condition like 2 h (1 h static/1 h dynamic) at 20 MPa and 60 °C from *Cannabis sativa* roots. The flow was set to 3 mL/min with 10 vol % EtOH as a co-solvent. They found that compared to conventional solvent extraction methods using n-hexane (0.698 ± 0.078 mg/g DW) and ethanol (0.0709 ± 0.036% by wt DW), the supercritical CO_2_ combined with ethanol method has significantly less friedelin (0.0548% by wt DW, *p* < 0.05) yield [65].

Microwaves are non-ionizing electromagnetic (EM) waves in the electromagnetic spectrum between radio-frequency waves and infrared. The frequency 915 MHz is best for industrial applications due to its greater penetration depth, while 2450 MHz is used in domestic microwave ovens and extraction applications with a wide range of commercial applications [93].

Alves et al. [61] used ultrasound-assisted extraction (UAE) and pressurized-liquid extraction (PLE) methods for friedelin extraction from *Monteverdia aquifolia* leaves extracts and compared them with the conventional Soxhlet extraction (SOX) extraction method. They found the highest yield of 8.3% in SOX with ethanol (360 min and ±78 °C) with 6.6% in UAE (30 min, 50 °C, amplitude 80%, and solvent/biomass ratio 20 mL g^−1^) and 5.3% in PLE (25 min and 60 °C) with the same solvent. However, UAE and PLE produced better extracts with greater TPC and AA than traditional extraction and used less solvent [61].

Mishra et al. (2020) [94] used microwave-assisted extraction (MAE) with eight vessels fitted with the exhaust in a closed extraction chamber accommodation. The friedelin concentrations of 0.010% by wt were extracted using fixed parameters like extraction vessel IR temperature limit (180 °C), pressure limit (20 psi), oscillation (ON), irradiation time (3 min), optimized microwave power (240 W), powdered drug (100 mg), and solvent (10 mL chloroform) with silicon carbide beads [94].

### 4.2. Quantification of Friedelin

Pharmaceutical researchers have studied friedelin extensively. Analytical quantification of friedelin is increasingly dependent on diverse methods. GC-MS and GC-FID, which enable new technology, are the main analytical methods.

Friedelin was detected in *Putranjiva roxburghii* Wall leaf extract (0.003% *w*/*w*), bark (0.04%), Ayurvedic formulation Femi-forte tablets (formulation 1) containing extracts of *Putranjiva roxburghii* (40 mg each tablet) (0.002%), and Femiplex tablets (13.05 mg each tablet) (formulation 2) (0.035%) [41]. Friedelin has an excellent linearity relationship (100–500 ng) with an r^2^ value of 0.9892. Friedelin had 32.15 and 97.44 ng/band detection and quantitation limits. The devised approach had interday and intraday precisions of 0.78% and 0.9%, respectively. Recovery experiments at three concentration levels showed 98.55% friedelin recovery [41].

Friedelin isolated from the cork of *Quercus suber* L. and cork byproduct was quantified by gas chromatography-mass spectroscopy (GC-MS) [95,96]. The chromatographic conditions were as follows: isothermal temperature 80 °C for 5 min followed by 285 °C for 15 min; 250 °C injector temperature; 285 °C transfer line temperature; 1:50 split ratio. The MS obtained data at 1 scan s−1 over m/z 33–800 in electron impact mode with 70 eV electron impact energy and the ion source were 200 °C. GC-MS quantification showed cork byproduct friedelin yields up to 1.4–5.0 g/kg [95] and *Quercus suber* cork yield friedelin of 2.47 g/kg dry weight [96].

Gas chromatography with flame ionization detection (GC-FID) was used for the quantification of friedelin extracted from *Maytenus ilicifolia* [97]. In this technique, 0.44 mg L^−1^ friedelin was quantified with the condition split mode (1:90) injected 1.0 L of samples in a 280 °C injector and 320 °C FID detector, with the column set at 300 °C isothermal mode, and the carrier gas was 1.5 mL/min helium [97].

## 5. Biological and Pharmacological Properties

Herbal therapies for the treatment of various diseases like diabetes, cancer, and liver problems are growing worldwide. Ayurvedic medicine uses herbal plant extracts to reduce the harmful side effects of medicines and improve efficacy. Numerous studies have demonstrated that Friedelin displays a diverse array of biological and pharmacological characteristics, encompassing hepatoprotective, antioxidant, anti-inflammatory, anticancer, anti-ulcerogenic, and neuroprotective properties.

### 5.1. Antioxidant and Hepatoprotective Activity

**Figure 4:**
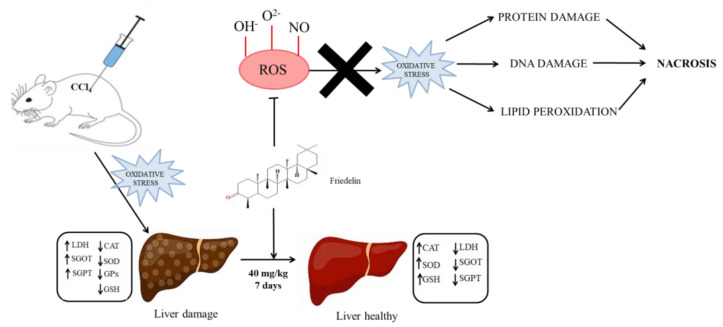
Antioxidant and hepatoprotective effect of friedelin.

Under physiological conditions, the body maintains a balance between the production and elimination of free radicals. Excessive free radicals damage cellular proteins, membrane lipids, and nucleic acids, causing lipid peroxidation [98]. The antioxidant and hepatoprotective activity of friedelin is shown in Figure 4. Friedelin isolated from *Azima tetracantha* Lam. leaves sowed a free radical scavenging effect on 2,2-diphenyl-picrylhydrazyl (DPPH), nitric oxide, hydroxyl, and superoxide radical with IC_50_ values of 21.1 mM, 22.1 mM, 19.8 mM, and 21.9 mM, respectively [99]. Friedelin (25 μg/mL) isolated from *Holothuria scabra* showed 90.22 ± 0.15% DPPH free radical scavenging activity with an effective concentration (EC_50_) value that was found to be 14.63 ± 0.01μg/mL [19]. Friedelin isolated from the ethyl acetate extract of the stem of *Tapinanthus bangwensis* showed 73.69% free radical scavenging activities comparable to 93.96% by ascorbic acid [20].

Sunil et al. in 2021 [21] evaluated the hepatoprotective effects of friedelin in CCl_4_-induced oxidative stressed rats by administering a dose of 40 mg/kg for a period of 7 days. The results showed that friedelin was able to restore this hepatic enzyme to normal levels and exhibited similar hepatoprotective effects to silymarin (25 mg/kg), indicating its significant antioxidant and hepatoprotective properties [21,99]. Friedelin fulfilled Lipinski’s rule of five and showed better bioactivity than Silibinin. The findings of this investigation indicated that the bioactivity score of friedelin is superior to that of Silibinin, a potent hepatoprotective medication [100].

### 5.2. Anti-Ulcerogenic Activity

**Figure 5:**
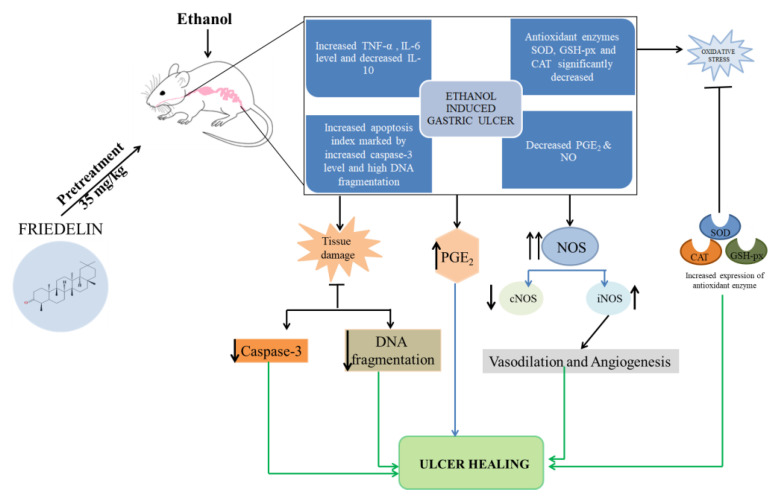
Schematic diagram of anti-ulcerogenic activity of friedelin.

Due to side effects from conventional medications, herbal remedies for gastrointestinal diseases are becoming more popular globally [101]. Antonisamy et al. [14] reported the anti-ulcerogenic activity of friedelin in an ethanol-induced gastric ulcer mice model (Figure 5). They found that pretreatment of 35 mg/kg friedelin exhibited a protective effect against harmful impact in ethanol-induced gastric ulcer mice. Friedelin lowered vascular permeability, pro-inflammatory cytokines [tumor necrosis factor-α (TNF-α) and Interleukin 6 (IL-6)], iNOS, caspase-3, and apoptosis, whereas anti-inflammatory cytokines (Interleukin-10) and gastric mucus increased [14].

By comprehending network theory and systems biology, network pharmacology is the future drug discovery paradigm. Shi et al. [102] were able to identify the top 10 targets coming from the protein–protein interaction network by molecular docking of friedelin with ulcerative colitis (UC) receptors (Figure S1). Further, they demonstrated that 42 mg/kg/d intra-peritoneal friedelin (i.p) alleviated the effects of colitis by lowering inflammatory cytokines (IL-1β and IL-6), increased anti-inflammatory cytokines (IL-10), restored colon mucosa, and improved symptoms and bodily function in a mice model induced by dextran sulfate sodium [102].

### 5.3. Antidiabetic Activity

**Figure 6:**
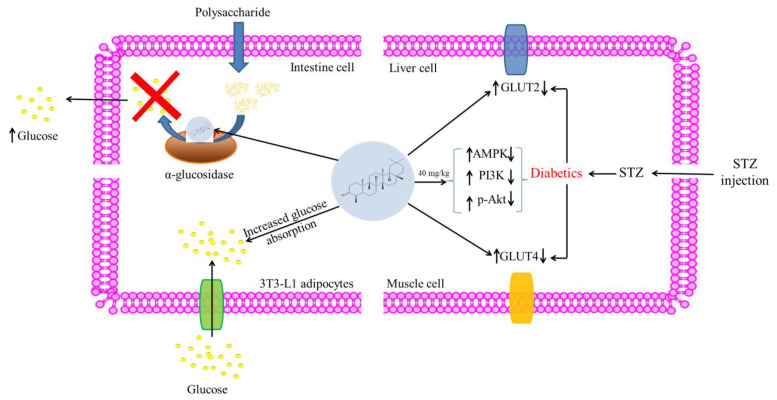
Schematic diagram of antidiabetic activity of friedelin.

Different mechanisms of antidiabetic effects of friedelin are shown in Figure 6. Susanti et al. [27] investigated the antidiabetic properties of friedelin isolated from twigs of *Garcinia prainiana* in insulin sensitivity 3T3-L1 adipocytes. The study observed that the intracellular fat accumulation was increased by 2.02-fold in the presence of friedelin when treated with an adipogenic cocktail (0.5 mM 3-isobutyl-1-methyl-xanthine (IBMX), 0.25 mM dexamethasone, 1 µg/mL insulin) compared to the cells treated with the vehicle. Adipocyte insulin sensitivity was tested using a deoxyglucose uptake assay. Compared to insulin-treated cells, friedelin increased glucose absorption by 1.8-fold [27].

Sunil et al. [21] used STZ-induced diabetic Wistar rats to show the antidiabetic mechanism of isolated friedelin from *A. tetracantha*. Diabetic rats had decreased protein expression of liver PI3K, p-Akt, GLUT2 and AMPK, and skeletal muscle GLUT4. GLUT2, PI3K, AMPK, p-Akt, and GLUT4 protein expressions decreased in diabetic rats. They found that 40 mg/kg friedelin increased PI3K, p-Akt, GLUT2, and AMPK protein expression in STZ-induced diabetic rats [21].

α-glucosidase hydrolyzes polysaccharides to glucose. Diabetes medicines target α-glucosidase because only the intestinal tract is capable of absorbing monosaccharides from the digestion of the poly- and oligosaccharides. Acarbose, miglitol, and voglibose are approved α-glucosidase inhibitors. An investigation demonstrated that 100 μM friedelin isolated from *Ficus drupacea* leaves inhibited α-glucosidase by 20.1% [62]. Further, friedelin obtained from ethyl acetate fraction of *Antidesma bunius* bark exhibited greater α-glucosidase inhibitory activity with IC_50_ 19.51 μg/mL compared to miglitol [28].

A computational approach to uncover the interaction between molecules extracted from *Syzygium cumini* and antidiabetic targets was done by Smruthi et al. [103]. Twenty-two phytoconstituents were docked with carbohydrate metabolism enzyme α-amylase using Autodock software (https://autodock.scripps.edu/ accessed on 19 November 2023) and the Lamarckian genetic algorithm. Friedelin had a significantly lower binding energy of −9.54 kcal/mol than the synthetic medication acarbose, which had a binding energy of −2.43 kcal/mol [103].

### 5.4. Anticancer Activity

**Figure 7:**
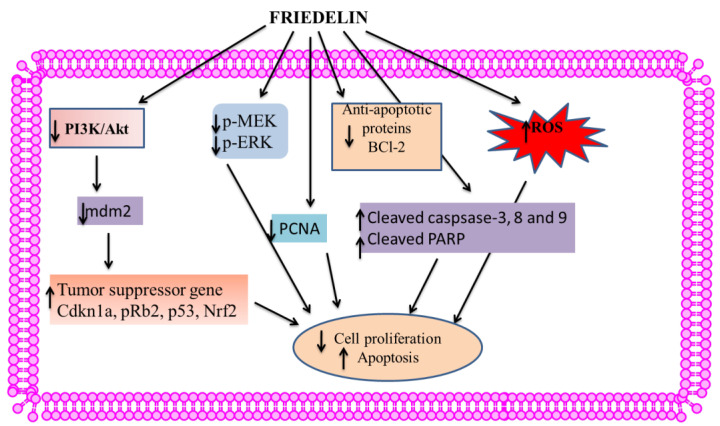
Schematic diagram of anticancer activity of friedelin.

Several clinical trials have shown that herbal remedies have anticancer properties [104]. Herbal medication was combined with conventional chemotherapy in anticancer therapy studies to improve therapeutic benefit, quality of life (QoL), and adverse effects [105]. Friedelin demonstrated anti-proliferative properties against various cancer cell lines [106]. The anticancer activity of friedelin is shown in Figure 7, and IC_50_ values are listed in Table 3.

Malignant breast cancer affects 18% of the world’s population and is the second leading cause of mortality for women [15,76]. An upregulation of estrogen receptors has been observed in several instances of breast cancer. Friedelin extracted from *Hopea odorata* demonstrated a −4.710 docking score with estrogen receptor alpha (ER-α) [76]. An in vitro cytotoxicity study of friedelin in human breast cancer cells (MCF-7) showed dose- as well as time-dependent inhibition of breast cancer proliferation [15]. Friedelin had an IC_50_ value of 0.51 μg/mL after 48 h for MCF-7 without causing any cytotoxicity in Vero and V79 cells. Friedelin caused DNA damage with significantly increased ROS levels. After 48 h of 1.2 μM friedelin treatment, Cdkn1a, pRb2, p53, Nrf2, and caspase-3 were upregulated, while Bcl-2, mdm2, and PCNA were downregulated, confirming apoptosis [15].

Prostate cancer is the second most common cancer in men and the fifth leading cause of death [109]. It has been reported that drugs targeting CYP17A1, a cytochrome P450c17 inhibitor, slow prostate cancer progression. The first approved CYP17A1 inhibitor was abiraterone acetate. However, successful drugs have side effects and therapeutic resistance in prostate cancer [110]. Friedelin derived from *Cassia tora* has been identified as the most optimal inhibitor of CYP17A1. Friedelin exhibits a stable binding pattern to the conserved binding pocket of CYP17A1, with a higher binding affinity compared to the control compound Orteronel. Friedelin’s IC_50_ was 72.025 and 81.766 μg/mL in hormone-sensitive (22Rv1-a human prostate carcinoma epithelial cell lines) as well as insensitive (DU145-a human prostate cancer cell lines) cell lines, respectively. The histopathological study confirmed that in animal trials, friedelin reduced prostate weight, blood PSA, prostate index, and testosterone [17].

Friedelin suppressed human leukemia cells (AML-196) while exhibiting minimal impact on healthy cells. The induction of apoptosis by friedelin was found to be associated with an increase in the expression of cleaved caspase-3, -8, and -9, as well as cleaved PARP. The levels of Bax protein were observed to be elevated, while those of Bcl-2 were found to be reduced. Friedelin inhibited AML-196 leukemia cell migration and invasion in transwell tests. Furthermore, Friedelin exhibited a dose-dependent inhibition of the MEK/ERK and PI3K/AT signaling pathways [16].

Using in silico molecular docking as well as molecular dynamics simulation, a total of 52 bioactive secondary metabolites from *Wedelia trilobata* were found to bind to the anti-apoptotic B-cell lymphoma-2 (Bcl-2) protein (PDB: 2W3L) structure. Friedelin’s binding energy against Bcl-2 protein was 10.1 kcal/mol compared to 8.4 kcal/mol for Obatoclax’s. In general, friedelin exhibits superior predicted absorption, distribution, metabolism, excretion, and toxicity (ADMET) properties compared to obatoclax. Friedelin derived from *W. trilobata* exhibits potential as an inhibitor of the Bcl-2 protein, which is known to be involved in cancer cell survival as well as resistance [18].

The cytotoxic effect of extracts and isolated compounds from *Elaeocarpus floribundus* was evaluated through a 3-(4,5-dimethylthiazol-2-yl)-2,5-diphenyl-2H-tetrazolium bromide (MTT) assay against human cervical (HeLa) as well as human T4 lymphoblastoid (CEM-SS) cancer cells. The friedelin demonstrated a strong inhibitory effect with an IC_50_ value of 3.54 ± 0.30 µg/mL toward HeLa cancer cells [47].

According to reports, friedelin exhibited cytotoxic properties towards the PC3 and U251 cancer cell lines. At 31 μM concentration, friedelin exhibited varying degrees of inhibition on K562, U251, and PC3 cancer cell lines of 0%, 25.8%, and 61.9%, respectively [111].

The Chinese Ministry of Health certifies bamboo shavings as useful food. Lu et al., (2010) examined the anticancer effects of triterpenoid-rich bamboo shavings extract (EBS) and friedelin. Friedelin separated from EBS was tested by MTT assay and showed strong anti-tumor activity on four cancer lines, L929 (IC_50_ value: 1.48 μg/mL), Hela (IC_50_ value: 2.59 μg/mL), A375 (IC_50_ value: 2.46 μg/mL), and THP-1 (IC_50_ value: 2.33 μg/mL), at an effected time of 48 h, compared to de-methylcantharidin [112]. 

Friedelin exhibited in vitro anticancer properties against glioblastoma multiforme (U87MG-GBM) cells. MTT assay indicates that FRI exhibited greater cytotoxicity towards U87MG cells in comparison to PRCC cells, as evidenced by IC_50_ values of 46.38 and 1271.77 µg/mL, respectively [11].

### 5.5. Neuroprotective Activity

Cognitive dysfunction is a major health issue in the 21st century, and many neuropsychiatric and neurodegenerative disorders, such as schizophrenia, Alzheimer’s disease dementia, seizure disorders, cerebrovascular impairment, and Parkinsonism, can severely debilitate [113]. Medicinal plant phytochemicals regulate the key inhibitory neurotransmitter receptors to maintain brain chemical homeostasis. Several plants cure cognitive problems in traditional medicine [114].

**Figure 8:**
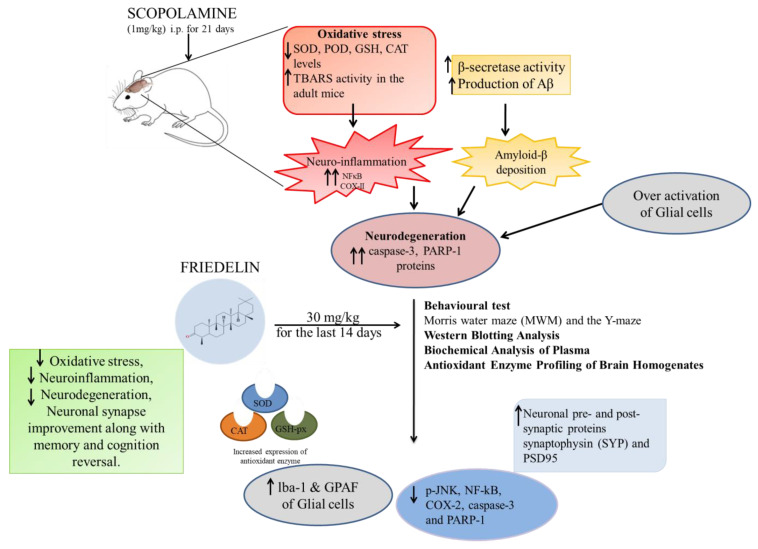
Schematic diagram of neuroprotective activity of friedelin.

Chang et al. (2013) examined the neuroprotective effects of 176 phytochemicals on primary cortical neurons after oxygen-glucose deprivation. Friedelin showed similar cell viability after oxygen-glucose deprivation (OGD) insult, which was 0.97 ± 0.003 at 1 μM and 1.00 ± 0.009 at 10 μM, compared to the untreated control group at 1.00 [22]. After OGD insult, friedelin exhibited neuroprotective effects, as shown in Figure 8.

Oxidative stress (OS) along with c-Jun N-terminal kinase (JNK) have been significant factors involved in neuroinflammatory signaling pathways as well as their associated neurodegenerative disorders. Sandhu et al. [23] tested the friedelin neuroprotection effect. Friedelin exhibited a protective effect against scopolamine-induced oxidative stress, neuro-inflammation, glial cell activation, and p-JNK as well as NF-κB and their downstream signaling molecules. Friedelin was found to enhance neuronal synapse and improve memory deficits induced by scopolamine through the inhibition of β-secretase enzyme (BACE-1) and amyloidogenic pathways [23] as shown in Figure 8.

### 5.6. Antimicrobial and Antiparasitic Activity

With the rise of microbe resistance toward various antimicrobial drugs, novel antimicrobial medicines developed from natural bioactive substances were sought [115]. Friedelin has been extensively researched for its potential health advantages and various biological properties, such as its ability to inhibit the growth of microorganisms. Friedelin extracted from *Pterocarpus erinaceous* [25], *Azima tetracantha* [24], *Jatropha tanjorensis* [26], *Calophyllum inophyllum*, *Maytenus undata* [74], *Calophyllum brasiliense* [73], *Garcinia smeathmannii* [72], and *Cola lateritia* K. Schum [116] have been reported to have antimicrobial properties against bacterial and fungal pathogens, as shown in Table 4.

The antibacterial and resistance-modifying activities of friedelin isolated from the methanol extract of *Paullinia pinnata* L. roots were evaluated in vitro against *Staphylococcus aureus* strains SA1199B, RN4220, and XU212. These strains possess the Tet (K), Nor (A), and Msr (A) transporters, which confer resistance to tetracycline, norfloxacin, and macrolides, respectively [71]. Friedelin had moderate antibacterial activity against three resistant *S. aureus* strains with MICs between 128 and 256μg/mL. At a concentration of 10µg/mL, friedelin did not exhibit any antibacterial activity. However, when combined with tetracycline, erythromycin, and norfloxacin, friedelin demonstrated a two-fold increase in potency [71].

Friedelin isolated from *Garcinia smeathmannii* exhibited good antimicrobial activity against *E. cloaclae*, *S. typhi,* and *S. faecalis* with MIC value 0.61 µg/mL [72]. Friedelin extracted from *Jatropha tanjorensis* in methanol solvent showed maximum antibacterial and antifungal activity [26]. At a concentration of 2.5 mg/mL, friedelin showed 40 mm, 40 mm, and 38 mm zone of inhibition toward Gram-negative bacteria *K. pneumoniae*, *P. mirabilis,* and *V. cholera*, respectively, 40 mm and 37 mm toward Gram-positive bacteria *B. cereus* and S. epidermis, respectively, and 31 mm and 33 mm toward fungi *A. fumigates* and *T. rubrum*, respectively [26]. However, friedelin isolated from *Maytenus undata* (MIC value >250 µg/mL) and *Calophyllum brasiliense* (MIC value >1000 µg/mL) was reported to have very little microbial activity [73,74].

Recently, the SARS-CoV-2 enzyme inhibitory potential of friedelin has been analyzed using in silico computational evaluation. It was reported that friedelin had more hydrogen bonds than remdesivir after 100 ns of molecular dynamic investigations and may be useful against the SARS-CoV-2 spike protein [117]. Friedelin formed a stable interaction with inflammatory cytokines IL-6 (−10.4 ± 0.02), IL-1β (−10.8 ± 0.01), and anti-inflammatory cytokines IFN-γ (−10.1 ± 0.01), thus protecting the pathogenicity of SARS-CoV-2 [118]. Friedelin extracted from *Vitex negundo* bound to five SARS-CoV-2 protein targets protease, spike glycoprotein, NSP3, NSP9, and NSP15 in which NSP9 and NSP15 showed the highest binding affinity of −9.6 Kcal/mol and −8.6 Kcal/mol, respectively [70].

Parasites have been with humans since the beginning of time and cause high morbidity and mortality, especially in developing nations. Despite recent advancements, parasitic disease control remains difficult. Friedelin is reported to exhibit trypanocidal [119], leishmanicidal [119,120], and antimalarial [121] properties. Friedelin has good antiplasmodial efficacy against *P. falciparum* strain K1 (IC_50_ = 7.70 μM) [122] and the chloroquine-resistant strain (W2) *P. falciparum* (IC_50_ of 7.20 ± 0.5 µM) [123].

## 6. Conclusions

This work presents detailed information on the various kinds of plant species that have been explored for the isolation of friedelin. Supercritical fluid extraction (SFE), ultrasound-assisted extraction (UAE), microwave-assisted extraction (MAE), and pressurized-liquid extraction (PLE) have been used to maximize friedelin extraction from plant sources. In this review, convincing evidence has been presented for the potent antioxidant activity of friedelin in several assay systems. Friedelin also suppresses inflammation in the brain tissues and regulates different cell signaling pathways. This review summarized that friedelin possesses potential as a valuable adjunctive therapy in the prevention and management of neurodegenerative disorders, cancer, diabetes, and inflammatory disease due to its natural origin. Nevertheless, it is important to note that the majority of the cited findings in this study are derived from experiments conducted in laboratory settings and animal models, which may not accurately reflect the impact on human subjects. Therefore, further investigation is required to explore the various pharmacokinetic parameters, potentially involving human participants, in order to ascertain the suitability of this substance as a prescribed medication in the future. High demand for friedelin has led to the development of CRISPR/Cas9 technology and gene overexpression plasmids to make it in genetically altered yeast. To efficiently acquire friedelin in large quantities, different plasmids must be studied.

## Figures and Tables

**Table 1:** Plant materials containing friedelin and solvent used for their extraction.

Plant Part	Plant	Family	Solvent Used for Extraction	References
Cork and/or stem barks	*Quercus cerris*	Fagaceae	Methanol, ethanol, dichloromethane, petroleum ether	[34]
	*Salix tetrasperma*	Salicaceae	80% aqueous methanol	[35]
	*Calophyllum pinetorum*	Clusiaceae	Sequentially with n-hexane and ethyl acetate	[36]
	*Drypetes tessmanniana*	Euphorbiaceae	Methanol	[37]
	*Prunus turfosa*	Rosaceae	5% benzene in chloroform	[38]
	*Pterocarpus erinaceus*	Fabaceae	Dichloromethane and methanol (1:1, *v*/*v*)	[39]
	*Terminalia avicennioides*	Combretaceae	Petroleum ether, ethyl acetate, chloroform, and methanol	[40]
	*Putranjiva roxburghii*	Euphorbiaceae	Chloroform	[41]
	*Endopleura uchi*	Humiriaceae	Hexane	[42]
	*Luehea ochrophylla*	Tiliaceae	Hexane and ethanol	[43]
	*Ancistrocarpus densispinosus* Oliv.	Tiliaceae	Methanol	[44]
	*Syzygium cumini* L.	Myrtaceae	70% methanol	[45]
	*Garcinia prainiana*	Clusiaceae	n-hexane	[27]
	*Uapaca ambanjensis*	Euphorbiaceae	Sequentially with n-hexane, dichloromethane, ethyl acetate, and methanol, respectively	[46]
	*Elaeocarpus floribundus*	Elaeocarpaceae	Sequentially with hexane, chloroform, ethyl acetate, and methanol	[47]
	*Elytranthe parasitica*	Loranthaceae	Methanol	[48]
	*Dombeya torrida*	Sterculiaceae	Chloroform	[49]
Leaves	*Azima tetracantha* Lam.	Salvadoraceae	Hexane	[50]
	*Maytenus ilicifolia*	Celastraceae	Hexane: Ethyl acetate (8:2, *v*/*v*)	[7,51]
	*Populus davidiana*	Salicaceae	Liquid WPM medium with 1% sucrose	[52]
	*Maytenus aquifolium*	Celastraceae	Ethanol	[53]
	*Garcinia imberti*	Clusiaceae	Hexane	[54]
	*Combretum duarteanum*	Combretaceae	Ethanol	[55]
	*Hibiscus tiliaceus*	Malvaceae	Dichloromethane	[56]
	*Vaccinium vitisidaea* L.	Ericaceae	Chloroform	[57]
	*Kalanchoe fedtschenkoi*	Crassulaceae	Hexane and chloroform	[58]
	*Grewia tiliaefolia*	Malvaceae	Methanol	[59]
	*Dombeya torrida*	Sterculiaceae	Dichloromethane: Methanol (50:50)	[49]
	*Garcinia rubroechinata*	Clusiaceae	n-hexane followed by methanol	[60]
	*Tapinanthus bangwensis*	Loranthaceae	Successively with n-hexane, ethyl acetate, and methanol	[20]
	*Monteverdia aquifolia*	Celastraceae	Ethanol	[61]
	*Ficus drupacea*	Moraceae	NA	[62]
Rhizomes	*Polygonum bistorta*	Polygonaceae	Chloroform	[63]
Flower	*Mammea siamensis*	Clusiaceae	Chloroform and methanol	[64]
Root	*Cannabis sativa*	Cannabaceae	EtOH and n-hexane	[65]
Aerial parts	*Prunus lusitanica*	Rosaceae	Petroleum ether	[66]
	*Leonotis nepetifolia* (L.) R. Br	Lamiaceae	Ethanol followed by methanol	[67]
Lichen	*Alectoria ochroleuca*	Parmeliaceae	Acetone	[10]
Moss	*Rhodobryum roseum*	Bryaceae	NA	[9]
Whole plant	*Solanum lyratum* Thunb	Solanaceae	Ethanol	[68]
Whole plant	*Euphorbia tirucalli*	Euphorbiaceae	Hexane and aqueous	[69]
	*Vitex negundo*	Lamiaceae	NA	[70]
	*Paullinia pinnata*	Sapindaceae	Methanol	[71]
	*Garcinia smeathmannii*	Clusiaceae	Methanol	[72]
	*Calophyllum brasiliense*	Clusiaceae	Methanol	[73]
	*Maytenus undata*	Celastraceae	Hexane, dichloromethane, acetone, and methanol	[74]
	*Calophyllum inophyllum*	Clusiaceae	Ethanol, butanol, chloroform	[75]
	*Jatropha tanjorensis*	Euphorbiaceae	Hexane, chloroform, and methanol	[26]
	*Wedelia trilobata*	Asteraceae	NA	[18]
	*Cassia tora*	Leguminosae	Ethanol	[17]
	*Hopea odorata*	Dipterocarpaceae	NA	[76]
	*Antidesma bunius*	Euphorbiaceae	Ethyl acetate	[28]
	*Azima tetracantha*	Salvadoraceae	Distilled water, phosphate buffer K_3_Fe(CN)_6_	[19]

NA—Not available.

**Table 2:** Extraction of friedelin using Soxhlet methods from different plant sources.

Plant	Plant Material	Extraction Condition	Solvent	Friedelin Concentration	References
*Quercus cerris*	Cork	120 mL solvent, 1 bar pressure, ~3 g biomass, 8 h.	Methanol	12.1 wt %	[34]
			Ethanol	15.2 wt %	
			Dichloromethane	23.7 wt %	
			Petroleum ether	41.3 wt %	
*Quercus cerris*	Cork	NA	Dichloromethane	26.03 wt %	[89]
*Maytenus aquifolium*	Leaves	10 g plant material with successive hexane and chloroform as an extraction solvent for 20 h each	Hexane	0.49 wt %	[90]
*Dombeya torrida*	Bark	1 kg of the stem bark powder extracted with chloroform for 48 h	Chloroform	NA	[49]
*Garcinia rubroechinata*	Leaves	500 g of the dry leaf powder extracted with n-hexane followed by methanol for 24 h	n-hexane followed by methanol	3.0 wt %	[60]
*Putranjiva roxburghii*	leaf and bark	50 g of dried powder extracted with Chloroform for 6 h.	Chloroform	0.003% *w*/*w* in leaf extract, 0.04% *w*/*w* in bark extract	[41]

NA—Not available.

**Table 3:** IC_50_ values toward different cell lines treated with friedelin.

S. No.	Cell Line	Cancer Type	Biological Source	Assay, Time of Execution	IC_50_ Concentration Used	References
**1**	MCF-7	Breast cancer	Human breast (adenocarcinoma)	MTT, 24 h, MTT< 48 h	0.76 μg/mL and 0.51 μg/mL 22.81 ± 2.1 µg/mL	[15,106]
**2**	22Rv1	Prostate cancer	Human prostate	MTT, 24 h	72.025 μg/mL	[17]
**3**	DU145	Prostate cancer	Human prostate	MTT, 24 h	81.766 μg/mL	[17]
**4**	AML-196	Leukemia	Human leukemia cells	CCK-8, 24 h	34 μg/mL	[16]
**5**	pADMSCs	Adipose-derived mesenchymal stem cells	Porcine mesenchymal stem cells	MTT, 48 h	15 μg/mL	[107]
**6**	U87 MG-GBM	Brain cancer	Human brain (glioblastoma astrocytoma)	MTT, 4 h	46.38 μg/mL	[11]
**7**	HeLa	Cervical cancer	Epithelioid cervix carcinoma	MTT, 72 h MTT, 24 h MTT, 24 h	3.54 ± 0.30 μg/mL 20.42 ± 2.3 μg/mL; 19.3 ± 1.27 μg/mL	[47,106,108]
**8**	Jurkat	Leukemia	Human blood (leukemic T-cell lymphoblast)	MTT, 24 h	29.15 ± 2.3 μg/mL	[106]
**9**	HT-29	Colon cancer	Human colon adenocarcinoma	MTT, 24 h	37.21 ± 3.61 μg/mL	[106]
**10**	T24	Urinary bladder cancer	Human bladder carcinoma	MTT, 24 h	12.81 ± 1.4 μg/mL	[106]
**11**	HSC-1	Squamous carcinoma	Human squamous carcinoma	MTT, 24 h	28.7 ± 1.98 μg/mL	[108]

**Table 4:** Antimicrobial activity of friedelin extracted from different plant sources.

Source of Friedelin	Method	Microorganism	Zone of Inhibition (mm)/Minimal Inhibition Concentration (µg/mL)	References
*Pterocarpus erinaceous*	1000 µg/mL disk diffusion	*Staphyloccocus aureus*	17	[25]
		*Aspergillus flavus*	10	
*Azima tetracantha*	Minimal inhibition concentration (MIC)	*T. mentagrophytes*	>250	[24]
		*T. simii*	125	
		*T. rubrum* 57/01	>250	
		*Epidermophyton floccosum*	125	
		*Scopulariopsis* sp.	>250	
		*Aspergillus niger*	125	
		*Curvularia lunata*	62.5	
		*Magnethophora* sp.	125	
		*Candida albicans*	>250	
*Jatropha tanjorensis*	2.5 mg/mL Disk diffusion	*Bacillus cereus* 430	40	[26]
		*Staphylococcus epidermis* 435	37	
		*Aeromonas hydrophila* 646	32	
		*Klebsiella pneumoniae* 432	40	
		*Proteus mirabilis* 425	40	
		*Proteus vulgaris* 426	17	
		*Salmonella paratyphi* 733	35	
		*Vibrio alcaligenes* 4442	27	
		*Vibrio cholera* 3906	38	
		*Aspergillus fumigates* 343	31	
		*Candida albicans* 227	NA	
		*Microsporum gyseum* 2819	NA	
		*Trichophyton rubrum* 296	33	
*Calophyllum inophyllum*	Disk diffusion	*Staphylococcus aureus*	6.6	[75]
		*Corynebacterium dptheriae*	3.5	
		*Salmonella typhi*	3.53	
		*Klebsiella pneumoniae*	4.0	
		*Proteus mirabilis*	3.11	
	% growth inhibition compared to standard drug miconazole and ketoconazole	*Pseudallescheria boydii*	81.04	
		*Candida albicans*	51.73	
		*Aspergillus niger*	85.09	
		*Trichophyton schoenleinii*	55.05	
*Maytenus undata*	MIC	*Staphyloccocus aureus*	>250	[74]
		*E. coli*	>250	
		*Pseudomonas aeruginosa*	>250	
		*Enterococcus faecalis*	>250	
		*Candida albicans*	>250	
		*Candida neofamans*	>250	
*Calophyllum brasiliense*	235 µM MIC	*Bacillus cereus*	>1000	[73]
		*Staphylococcus aureus*	>1000	
		*Staphylococcus saprophyticus*	>1000	
		*Streptococcus agalactiae*	>1000	
		*Enterobacter cloacae*	>1000	
		*Escherichia coli*	>1000	
		*Pseudomonas aeruginosa*	>1000	
		*Proteus mirabilis*	>1000	
		*Salmonella typhimurium*	>1000	
		*Candida albicans*	>1000	
		*Candida tropicalis*	>1000	
*Garcinia smeathmannii*	MIC	*E. cloaclae*	0.61	[72]
		*P. vulgaris*	1.22	
		*S. dysenteria*	1.22	
		*S. flexneri*	1.22	
		*S. typhi*	0.61	
		*S. typhimurium*	1.22	
		*B. megaterium*	1.22	
		*B. stearothermophilus*	1.22	
		*S. faecalis*	0.61	
		*C. albicans*	2.44	
		*C. krusei*	4.88	
		*C. gabrata*	2.44	

NA—Not available.

## Data Availability

Not applicable.

## Supplementary Materials

The following supporting information can be downloaded at https://www.mdpi.com/article/10.3390/molecules28237760/s1: Figure S1: Integrated targets between friedelin and ulcerative colitis (UC); Table S1: Computed chemical and physical properties of friedelin.

## Abbreviations

pOSCs	Oxido squalene cyclases
SHCs	Squalene-hopene cyclases
CoA	CoenzymeA
AACT	Acetyl CoA Cacetyl transferase
HMGCoA	3-hydroxy-3-methyl glutaryl CoA
MVA	Mevalonate
IPP	Isopentenyl pyrophosphate
DMAPP	Dimethyl allyl pyrophosphate
SFE	Supercritical fluid extraction
EM	Electromagnetic
UAE	Ultrasound-assisted extraction
PLE	Pressurized-liquid extraction
SOX	Soxhlet extraction
MAE	Microwave-assisted extraction
GC-MS	Gas chromatography mass spectroscopy
GC-FID	Gas chromatography with flame ionization detection
DPPH	2,2-diphenyl-picrylhydrazyl
TNF	Tumor necrosis factor-α
IL	Interleukin
UC	Ulcerative colitis
ER	Estrogen receptor
Bcl	B-cell lymphoma
ADMET	Absorption, distribution, metabolism, excretion, and toxicity
MTT	3-(4,5-dimethylthiazol-2-yl)-2,5-diphenyl-2H-tetrazolium bromide
EBS	Bamboo shavings extract
GBM	Glioblastoma Multiforme
OS	Oxidative stress
JNK	c-JunN-terminal kinase
MIC	Minimum Inhibitory Concentration
SARS-CoV	Severe Acute Respiratory Syndrome Corona Virus
IC	Inhibitory Concentration
IFN-γ	Interferon gamma
HSC	Hematopoietic stem cells
pADMSCs	Porcine adipose-derived mesenchymal stem cells
MCF-7	Michigan Cancer Foundation-7
CYP17A1	Cytochrome P450 family 17 subfamily A member 1
HT-29	Cells human colorectal adenocarcinoma cell lines
T24	Urinary bladder colorectal adenocarcinoma cell lines
AML-196	Acute myeloid leukemia 196
PRCC	Papillary renal cell carcinoma
