# Supplementary material for: Friedelin: Structure, Biosynthesis, Extraction, and Its Potential Health Impact
Source: Molecules. 2023 Nov 24;28(23):7760. doi: 10.3390/molecules28237760 (PMC10707989; doi:10.3390/molecules28237760)
Supplement: Supplementary file 1 [file molecules-28-07760-s001.zip › molecules-2708881-supplementary.pdf]

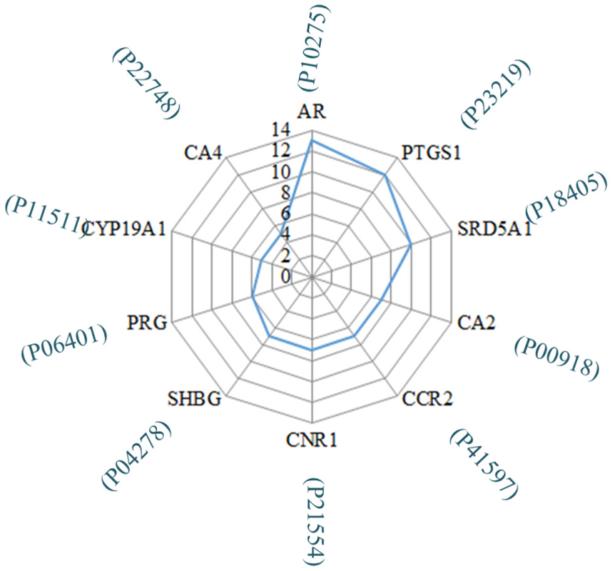

Figure S1. Integrated targets between friedelin and Ulcerative Colitis (UC). Abbreviation: AR-Androgen receptor; PTGS1-Cyclooxygenase-1; SRD5A1-Steroid 5-alpha-reductase 1; CA2-Carbonic anhydrase II; CCR2-C–C chemokine receptor type 2; CNR1- Cannabinoid receptor 1; SHBG Testis-specific androgen-binding protein; PGR-Progesterone receptor; CYP19A1-Cytochrome P450 19A1; CA4-Carbonic anhydrase IV.

Table S1. Computed chemical and physical properties of friedelin

S. No.	Property Name	Property Value
1	Molecular Weight	426.7g/mol
2	XLogP3-AA	9.8
3	Hydrogen Bond Donor Count	0
4	Hydrogen Bond Acceptor Count	1
5	Rotatable Bond Count	0
6	Topological Polar Surface Area	17.1Å^2
7	Heavy Atom Count	31
8	Formal Charge	0
9	Complexity	781
10	Isotope Atom Count	0
11	Defined Atom Stereocenter Count	9
12	Covalently-Bonded Unit Count	1
13	Compound Is Canonicalized	Yes
14	Appearance	Colorless white crystal
